# Memantine loaded PLGA PEGylated nanoparticles for Alzheimer’s disease: in vitro and in vivo characterization

**DOI:** 10.1186/s12951-018-0356-z

**Published:** 2018-03-27

**Authors:** Elena Sánchez-López, Miren Ettcheto, Maria Antonia Egea, Marta Espina, Amanda Cano, Ana Cristina Calpena, Antoni Camins, Nuria Carmona, Amélia M. Silva, Eliana B. Souto, Maria Luisa García

**Affiliations:** 1 Department of Pharmacy, Pharmaceutical Technology and Physical Chemistry, Faculty of Pharmacy, University of Barcelona, 08028 Barcelona, Spain; 2 Institute of Nanoscience and Nanotechnology (IN2UB), Faculty of Pharmacy, University of Barcelona, 08028 Barcelona, Spain; 3 Networking Research Centre of Neurodegenerative Disease (CIBERNED), Instituto de Salud Juan Carlos III, Madrid, Spain; 4 Department of Pharmacology and Therapeutic Chemistry, Faculty of Pharmacy, University of Barcelona, 08028 Barcelona, Spain; 5 Department of Biology and Environment, School of Life and Environmental Sciences (ECVA, UTAD), University of Trás-os-Montes and Alto Douro, Quinta de Prados, 5001-801 Vila Real, Portugal; 6 Centre for Research and Technology of Agro-Environmental and Biological Sciences, University of Trás-os-Montes and Alto Douro, CITAB-UTAD, 5001-801 Vila Real, Portugal; 7 Department of Pharmaceutical Technology, Faculty of Pharmacy, University of Coimbra (FFUC), Polo das Ciencias da Saúde Azinhaga de Santa Comba, 3000-548 Coimbra, Portugal; 8 REQUIMTE/LAQV, Group of Pharmaceutical Technology, Faculty of Pharmacy, University of Coimbra, Coimbra, Portugal

**Keywords:** Memantine, Nanoparticles, Alzheimer’s disease, Brain targeting, APPswe/PS1dE9 mice, β-Amyloid plaques, bEnd.3, Astrocytes

## Abstract

**Background:**

Memantine, drug approved for moderate to severe Alzheimer’s disease, has not shown to be fully effective. In order to solve this issue, polylactic-*co*-glycolic (PLGA) nanoparticles could be a suitable solution to increase drug’s action on the target site as well as decrease adverse effects. For these reason, Memantine was loaded in biodegradable PLGA nanoparticles, produced by double emulsion method and surface-coated with polyethylene glycol. MEM–PEG–PLGA nanoparticles (NPs) were aimed to target the blood–brain barrier (BBB) upon oral administration for the treatment of Alzheimer’s disease.

**Results:**

The production parameters were optimized by design of experiments. MEM–PEG–PLGA NPs showed a mean particle size below 200 nm (152.6 ± 0.5 nm), monomodal size distribution (polydispersity index, PI < 0.1) and negative surface charge (− 22.4 mV). Physicochemical characterization of NPs confirmed that the crystalline drug was dispersed inside the PLGA matrix. MEM–PEG–PLGA NPs were found to be non-cytotoxic on brain cell lines (bEnd.3 and astrocytes). Memantine followed a slower release profile from the NPs against the free drug solution, allowing to reduce drug administration frequency in vivo. Nanoparticles were able to cross BBB both in vitro and in vivo. Behavioral tests carried out on transgenic APPswe/PS1dE9 mice demonstrated to enhance the benefit of decreasing memory impairment when using MEM–PEG–PLGA NPs in comparison to the free drug solution. Histological studies confirmed that MEM–PEG–PLGA NPs reduced β-amyloid plaques and the associated inflammation characteristic of Alzheimer’s disease.

**Conclusions:**

Memantine NPs were suitable for Alzheimer’s disease and more effective than the free drug.

**Electronic supplementary material:**

The online version of this article (10.1186/s12951-018-0356-z) contains supplementary material, which is available to authorized users.

## Background

Alzheimer’s disease (AD) is the most prevalent neurodegenerative disorder amongst patients over 65 years old [1]. Memantine hydrochloride (MEM), a low-affinity voltage-dependent uncompetitive antagonist to glutamatergic *N*-methyl-d-aspartate (NMDA) receptors, is the only drug approved both in Europe and in the United States for moderate and severe degrees of the disease.

The clinical applications of nanoparticles (NPs) have proven great advantages for targeting and drug delivery, in particular, for the management of AD since current therapeutic strategies are compromised by the tight junctions and endothelial cells of the blood–brain-barrier (BBB) [2]. Nanoparticles, with an average size below 200 nm, may represent an alternative for prolonged drug delivery across the BBB, given their capacity for endocytic transport [3, 4]. While a number of polymers have already been used in the production of NPs, polyesters such as poly d,l-(lactic-*co*-glycolic) acid (PLGA), have been extensively applied for controlled drug delivery, including brain targeting [5, 6]. PLGA, which has been approved by the Food and Drug Administration, is one of the most successful biodegradable polymers because it undergoes hydrolysis to produce lactic and glycolic acid, easily cleared from the body [7]. In addition, advanced drug delivery systems based on PLGA NPs have recently demonstrated to be potential alternatives for the treatment neurodegenerative diseases [8]. A limitation on the use of PLGA NPs in drug delivery is, however, their fast uptake and clearance from the reticuloendothelial system (RES). To overcome the RES clearance, surface coating of NPs with poly (ethylene glycol) (PEG) has been recommended, an approach that has demonstrated to reduce NPs’ clearance significantly in vivo [9]. In addition, it has also been proven that such surface coating may increase NPs targeting and uptake through the BBB [10]. Loading drugs in PEG-PLGA NPs with a matrix structure is expected to prolong their circulation half-life compared to non-coated PLGA NPs, due to the presence of mobile and flexible PEG chains on their surface.

Memantine (MEM) is a good candidate for drug loading. MEM is an uncompetitive (open-channel) NMDA receptor antagonist with low-to-moderate affinity, which binds preferentially to the NMDA receptor-operated cation channels, overlapping the site of Magnesium [11]. Therefore, MEM decreases the excessive glutamate which causes neuronal death on AD patients [11]. Although this drug was found to improve patients’ cognition, global functioning behaviour and stage of dementia in comparison to placebo groups, results obtained from meta-analysis of AD monotherapy translate its limited clinical benefits (i.e. the assessment scores were not statistically significant between treated and non-treated groups) [12]. In this sense, drug delivery systems would increase drug concentration on the target site probably enhancing it’s effects against AD. In addition, despite being well-tolerated, MEM requires daily administration by the patients which, combined with the poor drug compliance, may also reduce the rates of successful treatment.

A sustained release formulation, based on PLGA NPs for oral administration, has been proposed to assure that the drug remains on the target site until the next patients’ intake of the medicine. MEM–PEG–PLGA NPs are expected to contribute to a time-stable dose on the brain, prolonging drug release, reducing administration frequency and decreasing the adverse-side effects. Comparing to other routes, and for chronic treatment schedules, oral administration offers comfort and improves patient’s compliance. Recent studies have demonstrated the added-value of loading drugs in PLGA NPs to enhance their oral bioavailability [13, 14]. PEG surfacing PLGA NPs have enhanced mucus permeating properties, therefore contributing to increase the drug’s bioavailability after oral administration [15].

In the present work, we report the development of a physicochemical stable, sustained-release MEM–PEG–PLGA NPs formulation, for the treatment of AD. Developed MEM–PEG–PLGA NPs are reported to be a non-invasive approach for brain targeting of MEM, with minimal adverse-side effects. The physicochemical stability of MEM after loading in PLGA NPs has been characterized by drug-polymer interaction studies, and by in vitro release profile. Cell viability was studied and the in vitro transport across the BBB was mapped. Transgenic and non-transgenic mice were orally treated with MEM–PEG–PLGA NPs and compared with the results obtained after treatment with free drug solution. Brain and plasma drug concentrations were measured, whilst behavioural test and histological studies were undertaken to elucidate the therapeutic efficacy of MEM–PEG–PLGA NPs against free drug, for brain delivery.

## Methods

### Materials

PLGA-PEG Resomer^®^ RGP d 5055 was obtained from Boehringer Ingelheim, Germany and memantine (MEM) was purchased from Capotchem (Hangzhou, China). Water filtered through Millipore MilliQ system was used for all the experiments and all the other reagents were of analytical grade.

### Production and physicochemical characterization of nanoparticles

MEM loaded NPs were produced by a modified double emulsion method described elsewhere [16, 17]. Briefly, a predetermined amount of PLGA–PEG was dissolved in ethyl acetate (EA) forming the organic phase. Aqueous phase (w_1_) was obtained by dissolving MEM in deionized water. Sonication energy was applied to form the primary emulsion (w_1_/o). The w_1_/o emulsion was then dispersed in 2 ml of deionized water containing PVA. Secondary emulsion (w_1_/o/w_2_) was formed with ultrasound energy [18]. A volume of 2 ml of PVA (0.3%) were then added, under magnetic stirring, to stabilize the colloidal system. Solvent was evaporated and NPs were washed by centrifugation at 15,000 r.p.m. during 20 min. The loading of NPs with rhodamine followed the same procedure. Empty NPs were prepared using the same approach but without addition of drug into the inner water phase [19].

Mean average size (Z-AVE) and polydispersity index (PI) of NPs were determined by photon correlation spectroscopy (PCS) using a ZetaSizer Nano ZS (Malvern Instruments). Measurements were carried out by triplicate at 180° in 10 mm diameter cells at a temperature of 25 °C. Zeta potential (ZP) was calculated from electrophoretic mobility as described elsewhere [20, 21].

Drug concentration was determined indirectly. Previously to the analysis, the non-loaded drug was separated from NPs by filtration/centrifugation at 14,000 r.p.m. (Mikro 22 Hettich Zentrifugen, Germany) using an Amicon^®^ Ultra 0.5 centrifugal filter device (Amicon Millipore Corporation, Ireland). The encapsulation efficiency (EE) was calculated by the difference between the total amount of drug and the free drug, present in the filtered fraction, using Eq. (1):

1 $$ EE\left( \% \right) = \frac{{{\text{Total}}\,{\text{amount}}\,{\text{of}}\,\,{\text{MEM}} - {\text{Free}}\,\,{\text{MEM}}}}{Total\,amount\,of\,MEM} $$

The quantification of MEM was performed in multiple reaction monitoring (MRM) mode using an ion trap mass spectrometer equipped with an atmospheric pressure electrospray ionization ion source, positive mode. The HPLC system was an Agilent 1100 series (Agilent Technologies, USA) coupled with a Brucker Ion Trap SL (Brucker Daltonics GmbH, Germany). MEM was separated on a reversed phase column (Kinetex de 2.6 μm 50 × 2.1 (Phenomenex) using methanol 0.1% formic acid in water 55:45 (v/v) as mobile phase. The flow rate was 1 ml/min at 45 °C [22].

### Design of experiments

Design of experiment (DoE) was used to optimize the developed formulation. Series of independent parameters and their influence on NPs properties were studied, determining the effects and interactions between factors. The effect of a factor × (*E*_*x*_), was calculated using Eq. (2):

2 $$ E_{x} = \frac{{\sum {{\text{X}}\left( + \right) - \sum {{\text{X}}\left( - \right)} } }}{n/2} $$

where ∑X(+) stands for the sum of the factors at their highest level (+ 1), ∑X(−) is the sum of the factors at their lowest level (−1), and *n*/2 is the half of the number of measurements. Interactions between factors (factor 1: factor 2) were also calculated. To estimate an interaction between two factors, the effect of the first factor at the lowest level of the second factor has to be calculated and subtracted it from the effect of the first factor at the highest level of the second factor.

For the study of the sonication parameters (Table 1) and concentration compounds (Table 2) two independent full factorial designs were performed. The mean size (Z-AVE), PI and ZP of the NPs were studied and the effects and interactions between factors were calculated. According to the composite design matrix generated by Statgraphics Plus 5.1 software, a total of 16 experiments (8 factorial points, 6 axial points and two replicated center points) were required for each design. The studied experimental responses were the result of the individual influence and the interaction of the three independent variables.

**Table 1:** Values of the matrix of a factorial design of sonication parameters and measured responses

	Amplitude		1st sonication time		2nd sonication time		Z_av_ (nm)	PI	ZP (mV)	EE (%)
	Coded level	(%)	Coded level	(s)	Coded level	(s)				
*Factorial points*										
F1	− 1	25.0	− 1	20.0	− 1	120.0	390.4 ± 2.2	0.213 ± 0.039	− 6.73 ± 0.04	3.46
F2	1	35.0	− 1	20.0	− 1	120.0	249.7 ± 4.7	0.069 ± 0.022	− 6.33 ± 0.49	9.59
F3	− 1	25.0	1	40.0	− 1	120.0	184.6 ± 0.7	0.125 ± 0.023	− 6.43 ± 0.45	42.57
F4	1	35.0	1	40.0	− 1	120.0	227.0 ± 2.6	0.057 ± 0.019	− 6.72 ± 0.33	36.89
F5	− 1	25.0	− 1	20.0	1	240.0	243.0 ± 0.9	0.194 ± 0.012	− 6.72 ± 0.24	7.43
F6	1	35.0	− 1	20.0	1	240.0	248.1 ± 1.9	0.053 ± 0.037	− 6.48 ± 0.15	14.69
F7	− 1	25.0	1	40.0	1	240.0	258.7 ± 4.5	0.198 ± 0.011	− 6.35 ± 0.33	22.63
F8	1	35.0	1	40.0	1	240.0	206.4 ± 1.2	0.061 ± 0.045	− 6.67 ± 0.30	2.88
*Axial points*										
F9	*1.68*	*38.4*	*0*	*30.0*	*0*	*180.0*	*222.4 ± 2.4*	*0.033 ± 0.011*	*− 5.63 ± 0.37*	*39.12*
F10	− 1.68	21.6	0	30.0	0	180.0	162.6 ± 0.4	0.262 ± 0.012	− 6.83 ± 0.37	39.36
F11	0	30.0	1.68	47.0	0	180.0	226.7 ± 4.4	0.236 ± 0.011	− 6.49 ± 0.25	19.94
F12	0	30.0	− 1.68	13.0	0	180.0	196.8 ± 2.5	0.103 ± 0.056	− 6.47 ± 0.55	43.10
F13	0	30.0	0	30.0	1.68	281.0	239.8 ± 0.7	0.056 ± 0.020	− 5.77 ± 0.47	23.39
F14	0	30.0	0	30.0	− 1.68	79.0	382.6 ± 5.2	0.221 ± 0.011	− 5.93 ± 0.21	33.95
*Center points*										
F15	0	30.0	0	30.0	0	180.0	220.1 ± 5.6	0.059 ± 0.019	− 5.36 ± 0.03	24.01
F16	0	30.0	0	30.0	0	180.0	222.1 ± 3.6	0.062 ± 0.021	− 5.36 ± 0.11	23.23

Italic values correspond to the optimized formulation of MEM loaded NPs

**Table 2:** Values of the matrix of a factorial design of concentration parameters and measured responses

	c _PLGA_− _PEG_		c _MEM_		c _PVA_		Z_av_ (nm)	PI	ZP (mV)	EE (%)
	Coded level	(mg/ml)	Coded level	(mg/ml)	Coded level	(mg/ml)				
*Factorial points*										
F1	− 1	10	− 1	3	− 1	2.5	270.0 ± 2.4	0.081 ± 0.002	− 13.4 ± 0.93	50.45
F2	1	30	− 1	3	− 1	2.5	450.1 ± 6.6	0.306 ± 0.022	− 8.27 ± 0.16	60.84
F3	− 1	10	1	9	− 1	2.5	230.1 ± 3.2	0.034 ± 0.026	− 2.95 ± 0.23	98.98
F4	1	30	1	9	− 1	2.5	369.3 ± 3.4	0.287 ± 0.028	− 3.33 ± 0.13	98.76
F5	− 1	10	− 1	3	1	7.5	324.4 ± 3.8	0.188 ± 0.009	− 9.79 ± 0.27	57.74
F6	1	30	− 1	3	1	7.5	287.8 ± 4.6	0.139 ± 0.029	− 7.84 ± 0.25	72.07
*F7*	*− 1*	*10*	*1*	*9*	*1*	*7.5*	*177.9 ± 2.9*	*0.034 ± 0.030*	*− 3.81 ± 0.44*	*81.23*
F8	1	30	1	9	1	7.5	223.4 ± 0.6	0.063 ± 0.007	− 3.88 ± 0.17	84.87
*Axial points*										
F9	1.68	37	0	5	0	5	260.4 ± 1.6	0.102 ± 0.018	− 8.85 ± 0.53	66.08
F10	− 1.68	3	0	5	0	5	147.1 ± 0.7	0.032 ± 0.007	− 4.96 ± 0.17	69.52
F11	0	20	1.68	11	0	5	204.3 ± 2.5	0.081 ± 0.018	− 3.86 ± 0.27	52.84
F12	0	20	− 1.68	1	0	5	238.8 ± 0.7	0.077 ± 0.013	− 14.5 ± 0.45	55.33
F13	0	20	0	5	1.68	9.2	199.0 ± 2.1	0.071 ± 0.027	− 5.59 ± 0.19	57.27
F14	0	20	0	5	− 1.68	0.8	272.2 ± 2.1	0.103 ± 0.033	− 3.26 ± 0.19	65.65
*Center points*										
F15	0	20	0	5	0	5	213.1 ± 0.4	0.023 ± 0.024	− 6.03 ± 0.27	72.61
F16	0	20	0	5	0	5	211.7 ± 0.3	0.034 ± 0.023	− 5.96 ± 0.16	40.92

Italic values correspond to the optimized formulation of MEM loaded NPs

### Nanospheres characterization and interaction studies

Morphology of the optimized formulation of MEM loaded NPs was determined by transmission electron microscopy (TEM), performed on a JEOL 1010 microscope (Akishima, Japan). The physical state and chemical interactions between drug and polymers were studied by thermal and X-ray diffraction analyses. For the interaction studies, NPs were washed by centrifugation and dried to constant weight previous to carry out the analysis. MEM thermal properties were studied by thermogravimetric (TG) analysis and differential thermal analysis (DTA) on a TASC 414/3 (Netsch, Thermal Analysis). Temperature ranged from 25 to 600 °C at 10 °C/min and AlO_3_ pan was used as a reference. All experiments were carried out under nitrogen flow. Thermograms were obtained by differential scanning calorimetry (DSC) on a Mettler TA 4000 system (Greifensee, Switzerland) equipped with a DSC 25 cell. Samples were weighed (Mettler M3 Microbalance) in perforated aluminium pans and heated under a flow nitrogen at a rate of 10 °C/min. X-Ray diffraction (XRD) was used to analyse the amorphous versus crystalline status of the samples. Compounds were sandwiched between polyester films and exposed to CuK” radiation (45 kV, 40 mA, λ = 1.5418 Å) in the range (2θ) from 2° to 60° with a step size of 0.026° and a measuring time of 200 s per step. Fourier-transformed infra-red (FTIR) spectra of different compounds were obtained using a Thermo Scientific Nicolet iZ10 with an ATR diamond and DTGS detector.

### Storage stability

The stability of MEM–PEG–PLGA NPs stored at three different temperatures (4, 25 and 38 °C) was studied by light backscattering using Turbiscan^®^Lab operated at constant temperature. For this purpose, a glass measurement cell was filled with 20 ml of sample. The light source is a pulsed near infrared light and was received by a backscattering detector at an angle of 45° from the incident beam. Backscattering data were acquired once a month for 24 h at intervals of 1 h. In addition to this technique, morphometric parameters (Z-AVE, PI and ZP) were also measured.

### In vitro drug release

In vitro drug release of MEM–PEG–PLGA NPs was studied against free MEM in PBS, in a bulk equilibrium direct dialysis bag technique under sink conditions for 36 h (n = 6). Briefly, a volume of 5 ml of each formulation was placed directly into a dialysis bag (cellulose membrane, 12–14 kDa, size 3,20/32’’ diameter, Iberlabo) and each bag was placed on 150 ml of isotonic phosphate-buffered saline (PBS) 0.1 M, pH 7.4 at 37 °C. At predetermined intervals, 1 ml of sample was withdrawn from the stirred release medium and simultaneously replaced with 1 ml of fresh buffer at the same temperature. Akaike’s information criteria was determined as an indicator of the suitability of the model for a given dataset. The model associated to the smallest Akaike’s information criteria value is considered as giving the best fit of the set of data [23].

### Cell culture

Cells were thawed, grown, maintained and regularly observed under a microscope. Two cell lines were used, namely, mouse microvascular endothelial cells (bEnd.3) and astrocytes from brain rat cortex. Primary cultures of astrocytes were obtained from bank GAIKER-IK4 culture. The bEnd.3 cells were maintained in their specific culture medium, DMEM + 10% FBS [24]. Cells and corresponding culture medium were tempered at 37 °C, 1 ml of cells was diluted in 9 ml of medium and the cell suspension was centrifuged at 4 °C for 5 min at a speed of 130*g*. The supernatant was removed and the cells were re-suspended in culture medium. Cells were seeded in 75 cm^2^ flasks and kept in an incubator at 37 °C, an atmosphere of 5% CO_2_ and a relative humidity of 95%. Once removed and washed, the cells were seeded with fresh medium.

### Cytotoxicity studies

Alamar blue reduction was used as a parameter for cell viability. This assay is based on that viable and metabolically active cells reduce resazurin to resorufin, which is released into the culture medium. This conversion is intracellular, facilitated by oxidoreductases of mitochondrial, microsomal and cytosolic origin. In a toxic event, where a loss of cell viability and proliferation occurs, the cells that comprise the epithelial tissue lose the ability to reduce resazurin. Therefore, resazurin reduction ratio is directly proportional to the number of viable cells. Absorbance was determined at λ of 570 and 620 nm, reduced and oxidized form, respectively [24]. Data were analysed by calculating the percentage of Alamar blue reduction and expressed as percentage of control (untreated), as reported before [25].

### In vitro transport across the BBB

**Fig. 1:**
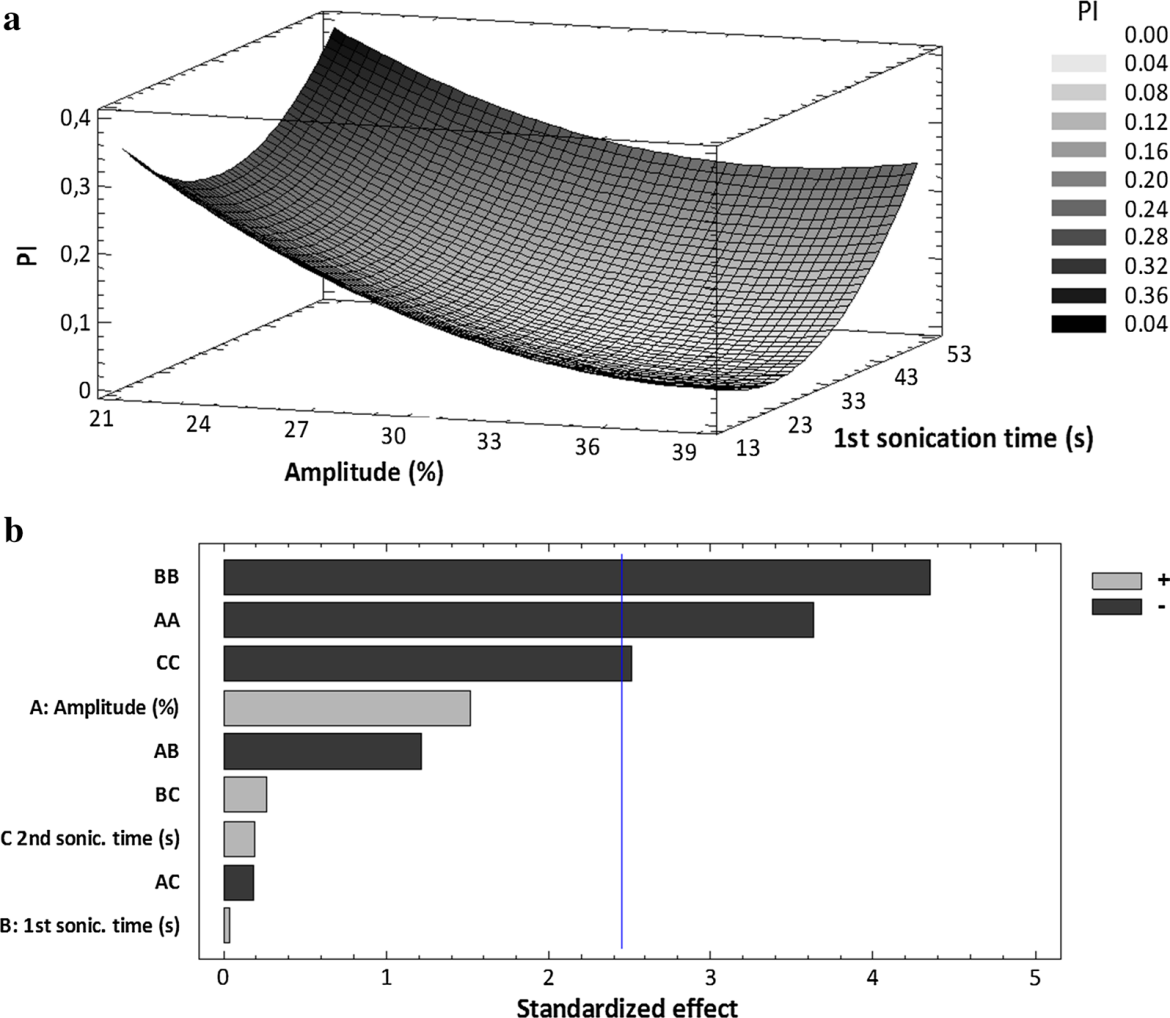
DoE of sonication parameters **a** surface plot of MEM-PLGA-PEG NPs PI and **b** Pareto’s chart of the effect of sonication parameters on ZP

In vitro BBB models have become a standard tool for estimating the ability of drugs to bypass the BBB at the early stage of drug development. In the present work, endothelial cell-based models were optimized by co-culturing the endothelial cells with astrocytes in Transwell systems, as shown in Fig. 1. Polycarbonate transwell inserts were used with a semipermeable membrane of 0.4 μm pore. For co-culture experiments, endothelial cells were seeded in the apical part of the inserts. A semipermeable filter was placed, and in the basolateral compartment cells from primary cultures of rat astrocytes were added.

### Trans-epithelial electrical resistance study

The brain vasculature is characterized by endothelial cells with strong tight junctions that limit paracellular diffusion of hydrophilic molecules selectively, according to their charge and size. When the movement of ions across the monolayer is restricted due to the proper functioning of the barrier, an electric potential gradient on both sides is generated. Transepithelial electrical resistance (TEER) is an indicator of cell confluence, monolayer integrity and the formation of tight junctions between cells. Thus, TEER manual measurements were taken daily until a steady state was reached, employing epithelial EVOM2 voltmeter connected to a pair of electrodes STX2. The system operates with two electrodes, which can be applied directly to the inserts. To calculate TEER of each insert, Eq. (3) was used and values are expressed in Ω cm^2^.

3 $$ TEER = \left[ {\varOmega_{cell\,monolayer} - \varOmega_{{Filter\,\left( {without\,cells} \right)}} } \right] \times \left[ {Filter\,Surface} \right] $$

Co-culture experiments were carried out in 24-well plates. Inserts were removed and placed in new media plates with Hanks and 0.5% bovine serum albumin (BSA). Apical media was removed, washed with Hanks and MEM–PEG–PLGA NPs were added (dissolved in 0.5% BSA Hanks) in the apical part of the inserts and left for 1 h. Furthermore, to verify that MEM–PEG–PLGA NPs did not compromise membrane integrity, a compound with low paracellular permeability, Lucifer yellow (LY), was added at the end of the study. Membrane integrity (with LY) was determined calculating the permeability coefficient by using the clearance principle which allows a permeability value independent of concentration.

### In vivo studies

Male APPswe/PS1dE9 (APP/PS1) and C57BL/6 mice were used for the in vivo studies. APP/PS1 animals co-express a Swedish (K594M/N595L) mutation of a chimeric mouse/human APP (Mo/HuAPP695swe), together with the human exon-9-deleted variant of PS1 (PS1-dE9), allowing these mice to secrete elevated amounts of human Aβ peptide [24]. The animals were kept under controlled temperature, humidity and light conditions with food and water provided ad libitum. Mice were treated in accordance with the European Community Council Directive 86/609/EEC and the procedures established by the *Departament d’Agricultura, Ramaderia i Pesca of the Generalitat de Catalunya*. Every effort was made to minimize animal suffering and to reduce the number of animals used. 6 month-old animals, divided into six groups, were used for the present study, with at least 10 WT and 10 APP/PS1 transgenic mice, per group. In each genotype group, the mice were treated either with untreated water, with free MEM or with MEM NPs (Additional file 1: Figure S1). Mice were treated for 2 months with MEM at therapeutic dose (30 mg/kg/day). MEM–PEG–PLGA NPs were orally administered in alternate days calculating the volume for each animal previously weighted followed by a regime of untreated water. After the in vivo testing, the animals were sacrificed. Prior to sacrifice, mice were left fasting for at least 8 h and in the case of treated mice. At least 6 mice in each group were used for histological studies [26].

### Nanoparticles brain distribution

To tackle the NPs uptake and visualize them in the target tissue in vivo, MEM NPs were fluorescently labelled by linking PLGA to Rhodamine. NPs were orally administered for 1 week on alternate days and after 24 h of clearance mice were anesthetized with sodium pentobarbital and perfused with 4% paraformaldehyde. Brains were stored at 4 °C overnight dehydrated in 30% phosphate-buffered sucrose solution. Samples were preserved at − 80 °C and coronal sections of 20 μm were obtained by a cryostat (Leica Microsystems, Wetzlar, Germany). Samples were visualized using a fluorescence microscope using Rhodamine filter (BX41 Laboratory Microscope, Melville, NY-Olympus America Inc).

Previously to the study of the therapeutic effects, drug at steady state levels was quantified. Blood samples were extracted from the facial vein and samples were centrifuged during 20 min at 2000 r.p.m. adding EDTA (10 μl K_2_EDTA 18 mg/ml) to avoid blood coagulation. Mice were sacrificed by cervical dislocation. Amantadine was added as internal standard and MEM extraction was carried out using organic solvents (*t*-butyl methyl ether and diethyl ether–chloroform for brain and blood samples, respectively). Solvents were evaporated under nitrogen flow and samples were reconstituted with methanol [27, 28]. Samples were analysed as described previously (section NPs production). The analyses were carried out using the parent to daughter combinations of *m*/*z* 180 > 163 (MEM) and *m*/*z* 152 > 135 (amantadine) [22].

### Morris water maze

The Morris water maze (MWM) test was conducted in a circular tank filled with water at 21 ± 2 °C and divided into four equal quadrants. A white platform was submerged below the water surface in the middle of the northeast quadrant. The behavioural data were acquired and analysed using a computerized video tracking system. The procedure of the behaviour assessment consisted of a six-day navigation testing session and a probe trail. Mice received five trials per day for 6 successive days continuing with the same drug regime. Animals were placed into the maze facing the tank wall at water-level. They were allowed to swim freely for 60 s to seek the invisible platform and allowed to remain there for 10 s. If a mouse failed to find the platform, it was guided to it and left there for 30 s. The probe trial was performed the day after the last training test. In the probe test, the hidden platform was removed, and the mice were released from the southwest quadrant and allowed to swim for 60 s. Results were calculated individually for each animal [29].

### Immunohistochemistry studies

Mice were anesthetized with sodium pentobarbital and perfused with 4% paraformaldehyde in 0.1 M phosphate buffer (PBS) after the probe trial. Brains were stored in 4% paraformaldehyde at 4 °C overnight then dehydrated in 30% phosphate-buffered sucrose solution for cryoprotection. Samples were preserved at − 80 °C and coronal sections of 20 µm were obtained by a cryostat (Leica Microsystems, Wetzlar, Germany). Sections were incubated overnight at 4 °C with the rabbit anti-GFAP (1:2000; Dako, Glostrup, Denmark) primary antibody, and sequentially incubated for 2 h with Alexa Fluor 594 goat anti-rabbit antibody at room temperature (1:500; Invitrogen, Eugene, OR, USA). Staining of β-Amyloid plaques was performed using Thioflavin S (ThS 0.002%, Sigma-Aldrich) to compare β-amyloid plaque density among different treatment groups. Sections were counterstained with 0.1 μg/ml Hoechst 33,258 (Sigma-Aldrich, St Louis, MO, USA) and rinsed afterwards with PBS 0.1 M [30]. ThS-stained β-amyloid plaques were visualized using a fluorescence microscope with a fluorescence filter (BX41 Laboratory Microscope, Melville, NY-Olympus America Inc). For each image, the proportion of total image area covered by fluorescently stained β-amyloid plaques was quantified. For each mouse, four fields per section with the highest density of plaques were chosen as representative and were averaged [31].

### Statistical analysis

All of the data are presented as the mean ± S.D. Two-way ANOVA followed by Tukey post hoc test was performed for multi-group comparison. Student’s *t* test was used for two-group comparisons. Statistical significance was set at p < 0.05 by using GraphPad Prism version 5.00 for Windows, GraphPad Software, San Diego California USA.

## Results and discussion

### Optimization of NPs’ parameters

Double emulsion evaporation method was chosen for the production of PLGA NPs due to its suitability for the loading of hydrophilic drugs, such as MEM. Since the mean particle size is a critical parameter to assure that NPs are absorbed in the gastrointestinal tract and achieve the BBB, the aim of the factorial design was to produce MEM–PEG–PLGA NPs with a mean size 100 and 200 nm. Since MEM is insoluble in ethyl acetate, this organic solvent has been used for the preparation of w_1_/o/w_2_ emulsions, allowing the retention of the drug in the inner aqueous phase. From preliminary studies, the addition of small amount of polyvinyl alcohol (PVA, 0.3%) after the second emulsification process was shown to contribute to the decrease of the mean size of NPs from 264.6 nm (with a bimodal distribution) down to 220.1 nm (with a monomodal distribution, PI < 0.1). These results were attributed to the delay of the solvent diffusion to the outer aqueous phase upon addition of the aqueous PVA solution, limiting the risk of droplets agglutination and polymer precipitation [19]. The results obtained from the full factorial designed performed for the selection of the appropriate sonication parameters (i.e. wave amplitude and sonication time) are shown in Table 1.

**Fig. 2:**
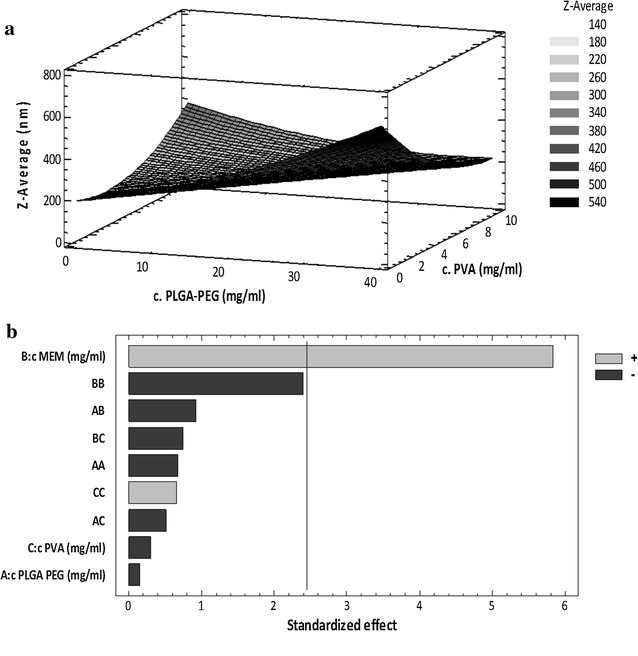
DoE of concentration parameters; **a** surface plot of MEM –PLGA-PEG NPs z-AVE and **b** effect of compounds concentration on ZP

Factorial design demonstrates that the interaction between 1st and 2nd sonication times increased the mean size of NPs. As a consequence, a lower 1st sonication time was chosen to obtain NPs smaller than 200 nm. High amplitudes, around 38%, are mandatory to obtain small and monodispersed NPs (Fig. 2a) [19]. As shown in Fig. 1b, amplitude was a significant parameter regarding particles surface charge, establishing a trend where increasing the amplitude causes a slight increase of the negative charge and, subsequently, the creation of more stable particles. Therefore, the maximum amplitude (F9, Table 1) would be applied.

**Fig. 3:**
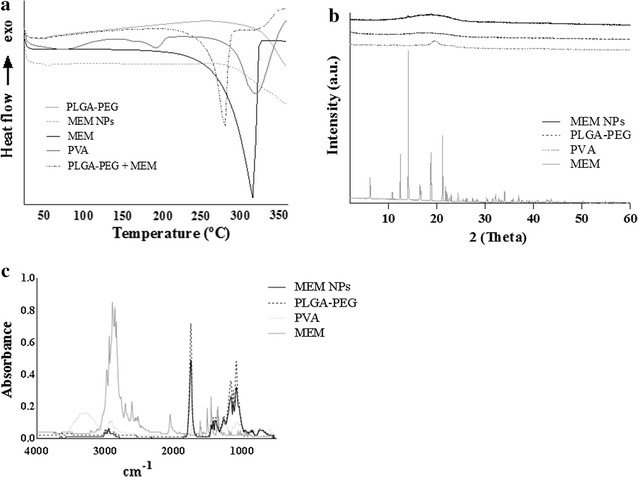
MEM-PLGA-PEG NPs interaction studies **a** differential scanning calorimetry analysis, **b** X-ray diffraction and **c** Fourier transformed infra-red analysis

The optimized concentration parameters of the formulation compounds have also been studied (Table 2). The increase of the polymer concentration caused the increase of both z-AVE and PI (Fig. 3a). Indeed, the higher the viscosity of the inner aqueous phase of the primary emulsion (w_1_/o), the less efficient is the reduction of the emulsion droplet size during the second emulsification step (w_1_/o/w_2_) [16, 17]. The higher the PVA concentration, the smaller the NPs obtained (Fig. 2a). These results suggest that the optimized PVA concentration should be able to ensure enough surfactant molecules to cover the interface between the organic phase and the external aqueous phase, improving the protection of the droplets from coalescence [17].

Pareto’s chart (Fig. 2b) shows that MEM concentration influenced the surface electrical charge of NPs significantly. MEM has an amine group which can easily be protonated and decrease negative surface charge caused by the polymer [32, 33]. However, while high MEM concentrations negatively affect the particles stability, a statistically significant relationship between EE and MEM concentration was established. According to the factorial design data, a suitable formulation was achieved with a minimum concentration of polymer (10 mg/ml), an upper-intermediate drug concentration (9 mg/ml) and a maximum amount of PVA (7.5 mg/ml) (F7, Table 2). After ultracentrifugation at 15,000 r.p.m. for 20 min of the optimized formulation, NPs kept their size properties (z-AVE of 152.6 ± 0.5 nm and PI 0.043 ± 0.009), although ZP was more negative (− 22.4 ± 0.5 mV), which was attributed to the removal of surfactant molecules from the surface of the particles. Detailed structure of MEM–PEG–PLGA NPs was further characterized by TEM, which confirmed the spherical shape and smooth surface of NPs (Additional file 1: Figure S1).

### Characterization of NPs and interaction studies

In vitro and in vivo drug release profiles are highly dependent on the physical state of the drug inside the NPs. TG and DTA were therefore used to study the interaction between MEM and polymers. MEM shown to be stable at low temperatures probably due to the presence of the anhydrous [33]. TG profile of MEM exhibited a weight loss starting at 290 °C, and finishing at 354 °C, which correspond to the complete degradation of drug. (Additional file 1: Figure S2). DTA showed an onset of endothermic event at 280 °C followed by a maximum at 352 °C being these results similar to those obtained by DSC. A thermal decomposition of MEM was shown to occur in two steps, corresponding the latter to a final oxidative degradation.

DSC curves of MEM, PVA, PEG–PLGA, MEM–PEG–PLGA NPs, and physical mixture of MEM are depicted in Fig. 3a. PVA exhibits two endothermic peaks, corresponding to the melting (192.86 °C) and decomposition (318.61 °C) events, respectively. PEG-PLGA onset transition temperature (T_g_) takes place around 44.50 °C. PLGA without PEG chains exhibited a T_g_ around 54.18 °C. The presence of PEG chains produced a decrease of the T_g_ values, attributed to the plasticizing effect based on the reduction of the attractive forces among the polymer chains. MEM displayed a melting transition followed by decomposition between 190 and 322 °C, exhibiting a thermal event comprising both phenomena. DSC analysis of MEM–PEG–PLGA NPs displayed an endothermic event corresponding to the T_g_ of the polymer occurring at 50.56 °C. The increasing of the T_g_ of the polymer has been attributed to the incorporation of an alkaline drug, which causes interactions between the carboxylic groups of the polymer.

Results from XRD studies are shown in Fig. 3b. Drug powder diffraction pattern showed sharp crystalline peaks, whereas PEG–PLGA showed an amorphous profile. MEM–PEG–PLGA NPs displayed a profile similar as PEG–PLGA, but a slight attenuated peak corresponding to the drug was also observed. The surfactant displayed a semi-crystalline pattern, not present in the formulation. This fact demonstrates the effectiveness of the centrifugation process, confirmed by FTIR analysis (Fig. 3c). This suggests that the surfactant acts only as adjuvant in the NPs production, stabilizing the freshly prepared particles while it is not entrapped in the polymer because it was effectively removed by centrifugation. This property is relevant since a high surfactant concentration may induce toxicity by establishing an interconnected network with the polymer [34].

FTIR analysis (Fig. 3c) does not show any evidence of chemical interaction or strong bond formation between MEM and PEG–PLGA or between NPs and surfactant. The stretching band of the polymer carbonyl groups (C=O) was observed at 1740 cm^−1^, whereas the first polymer bands are due to C–O PLGA–PEG bonds [35]. The bond at 2950 cm^−1^ clearly indicates the presence of C–H (ethylene glycol). PVA exhibits a number of absorption peaks at 2900, 1324, 843 and 1084 and 3237 cm^−1^ due to C–H stretching, C–H bending and C–O stretching, which are not depicted in the profile obtained for MEM–PEG–PLGA NPs. Around 3000 cm^−1^ MEM showed the amine corresponding peak associated with N–H stretching bond. As reported by other authors, the peak at 1648 cm^−1^ indicates presence of C–O group attached to –NH [36].

### Storage stability

**Fig. 4:**
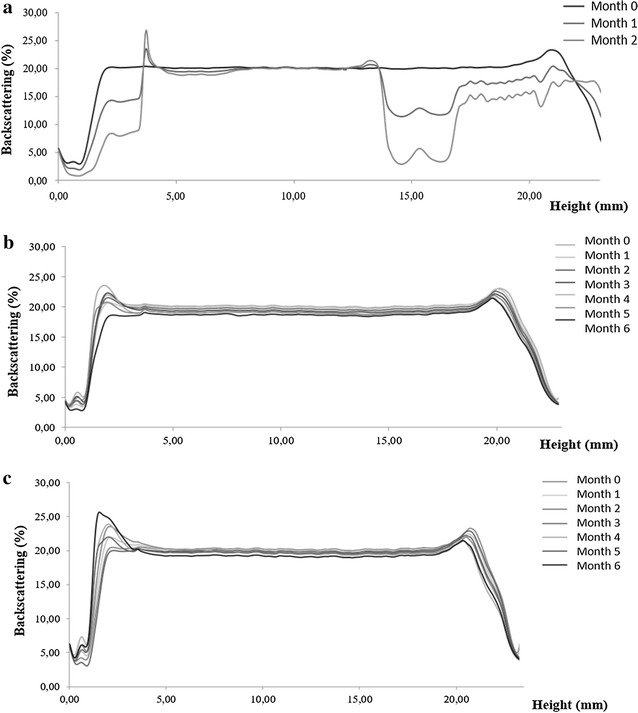
Backscattering profile of MEM-PLGA-PEG NPs stored for 6 months; **a** 38 °C, **b** 25 °C and **c** 4 °C

Stability of the developed NPs at different temperatures (4, 25 and 38 °C) was also monitored. Samples stored at 4 and 25 °C remained visually unchanged during the first 6 months of storage. Samples stored at 38 °C were completely transparent and unstable by the end of the 1st month because of the degradation of the polymer induced by higher temperatures (Fig. 4a).

Samples stored at 4 and 25 °C kept their ZP and size parameters (z-AVE and PI) for 6 months. No statistically significant differences were found between formulations stored at 4 and at 25 °C. Backscattering profiles at both temperatures were similar to those obtained by the end of the 1st month, but NPs stored at 25 °C showed a slight decrease of the light scattered percentage corresponding to the bottom of the sample, which was not observed at 4 °C (Fig. 4b, c). This result was attributed to a slight NPs sedimentation process being preferential the particles’ storage at 4 °C.

### In vitro drug release

**Fig. 5:**
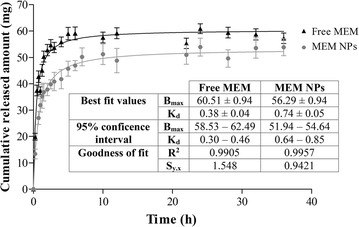
*In vitro* release profile of MEM from PBS solution or MEM-PLGA-PEG NPs. Mean parameters were obtained adjusting data to hyperbola equation

In vitro drug release was analysed against a drug solution in PBS (free MEM). Free MEM release was faster than the observed for MEM–PEG–PLGA NPs (Fig. 5). The optimized formulation showed an immediate release (*burst* release) attributed to the non-loaded MEM fraction which is weakly bound to the NPs’ surface, because of the PEG coating [37]. After this initial phase, the drug displayed a sustained release diffusing slowly from the polymeric matrix into the release medium. Akaike’s information criterion for hyperbola adjustment was 64.97 for MEM-loaded NPs and 86.8 for free drug. Parameters corresponding to hyperbola adjustment were analysed. K_d_, equilibrium dissociation constant, expressed in concentration, corresponding to MEM–PEG–PLGA NPs was almost twice (0.74 for drug loaded NPs and 0.38 for the free drug) than K_d_ obtained for the free drug, confirming the slower release of the drug from the colloidal system.

### Cytotoxicity studies

**Fig. 6:**
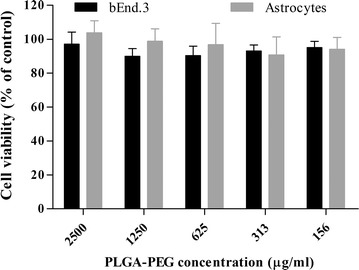
Cell viability assessment using Alamar blue on brain cell lines

Cell viability of MEM–PEG–PLGA NPs was measured in bEnd.3 (brain endothelial cells) and rat astrocyte primary cultures. These cells form the BBB and, for this reason together they are considered as a suitable model to test nanoparticles cytotoxicity. Following the incubation for 24 h, MEM–PEG–PLGA NPs did not show any measurable toxic effect (Fig. 6). These results confirm that the developed particles are biocompatible with both endothelial glial brain cells. In addition, the slight amount of PVA that could remain after centrifugation process did not induce any toxicity nor influenced the normal growth of both epithelial cell lines within the assessed doses.

### In vitro and in vivo transport across the BBB

**Fig. 7:**
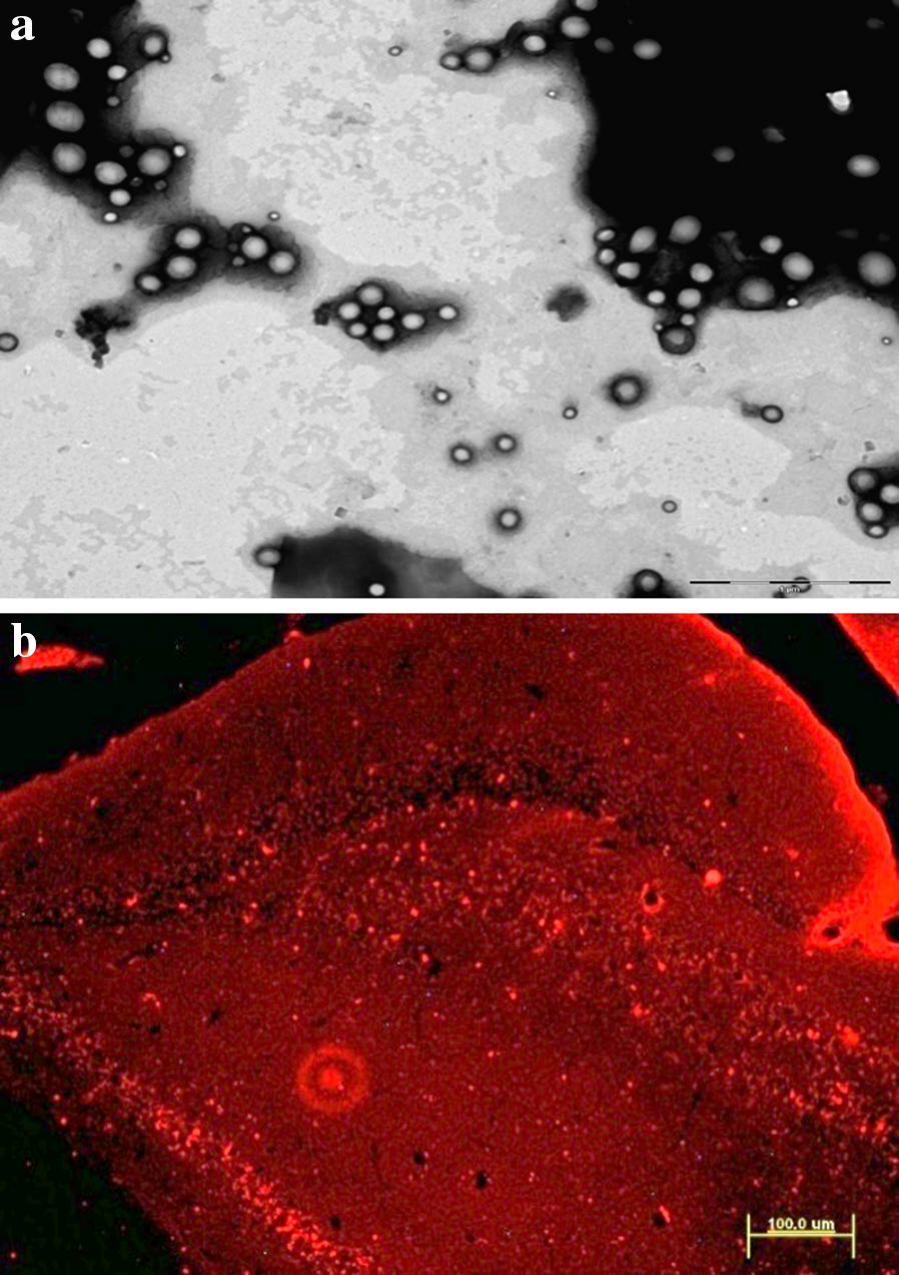
**a** TEM pictures of MEM–PEG–PLGA NPs on the basolateral compartment of the BBB transport model after one hour of incubation, **b** Hipocampus of WT mice with PLGA-Rho NPs

Results show that 40% of the initial MEM–PEG–PLGA NPs were retained by the cell membrane of the in vitro model within 1 h of incubation, whereas only 30% of the initial MEM was found inside the barrier. Drug permeability coefficient (P_e_) in this model was 0.933. This fact indicates that NPs retained in this tissue would be able, either to achieve a slow drug release from there, or to partially cross through it and release the drug into the basolateral media. TEM images demonstrate that the part of MEM–PEG–PLGA NPs achieving the BBB remained spherical and non-aggregated with an average size below 200 nm (Fig. 7a). In addition, Lucifer Yellow (LY) was used as control at the end of the study, showing that these systems do not cause disruption of the BBB as no increase of paracellular passage of LY was observed (P_e_ < 1 in all the experiments). In addition, other authors that develop similar drug delivery systems for CNS diseases also demonstrate that the NPs were able to effectively transport the drugs. Year ago, Alyautdinvand colleagues developed Polibutylcianoacrilate NPs encapsulating Loperamide (which is unable to pass through the BBB), showing that the drug delivery systems (around 290 nm and PI below 0.1) were effective [38]. More recently, Liu and co-workers developed PEGylated NPs to cross the BBB with an average size smaller than 200 nm, able to cross the BBB [39]. The author’s labelled the NPs fluorescently obtaining qualitative results and no disruption of the BBB was reported. Hereby, we provide behavioral and molecular data that gives solid evidence of the enhancement of the drug activity provided by the NPs [39]. In addition, spherical shape demonstrated to increase their transport across the BBB [40]. Moreover, grafting NPs with PEG decreases protein adsorption and slows down the nanomaterials clearance improving blood circulation time; as a consequence, PEGylated NPs accumulate more efficiently in the brain [41].

As can be observed in Fig. 7b, Rhodamine labelled NPs were able to be absorbed by the GI, cross the BBB and reach the brain upon oral administration. Oral delivery is the most common route of administration and NP drug carriers that can shield drugs from degradation and deliver them enabling more efficient and sustained drug delivery [42]. Particles synthesized from commonly used polymers, such as PLGA, may achieve mucoadhesion via hydrogen bonding, polymer entanglements with mucins, hydrophobic interactions, or a combination of these mechanisms [42]. The advantage that offer the entrapment on polymeric NPs has been demonstrated by several authors. For instance, Mittal et al. developed similar drug delivery systems to be administered orally and demonstrated their ability to be absorbed by the GI and provide therapeutic blood levels [43]. It has also been demonstrated that, in addition of NPs size, the solvent and the stabilized used has an effect on drug release. Other authors, such as Sahana and co-workers develop similar drug delivery systems with a mean size around 270 nm using ethyl acetate and PVA obtaining a prolonged drug release for 3 days after their administration and Kalaria and collegues also achieve superior effectivity of oral PLGA NPs compared with the free drug in vivo [44, 45]. Moreover, NP surface characteristics can be tailored to optimize mucoadhesion [42]. As demonstrated by Tobío and co-workers, PEGylation of polymeric NPs of a mena size around 160 nm, imparts additional protection against enzyme induced aggregation and degradation in simulated GI fluids in vitro [46]. Previous publications of our group demonstrated that PLGA PEGylated NPs were suitable for GI absortion [47] showing the NPs groups and increased behavioral and molecular effectivity.

In addition, NPs demonstrated to be able to reach the hippocampus confirming that this drug delivery systems could be an effective strategy to achieve an increased drug release. Furthermore, plasma levels after a single NPs dose were recorded achieving MEM concentrations in plasma around 883.02 ng/ml after 40 h of clearance.

### Morris water maze test

**Fig. 8:**
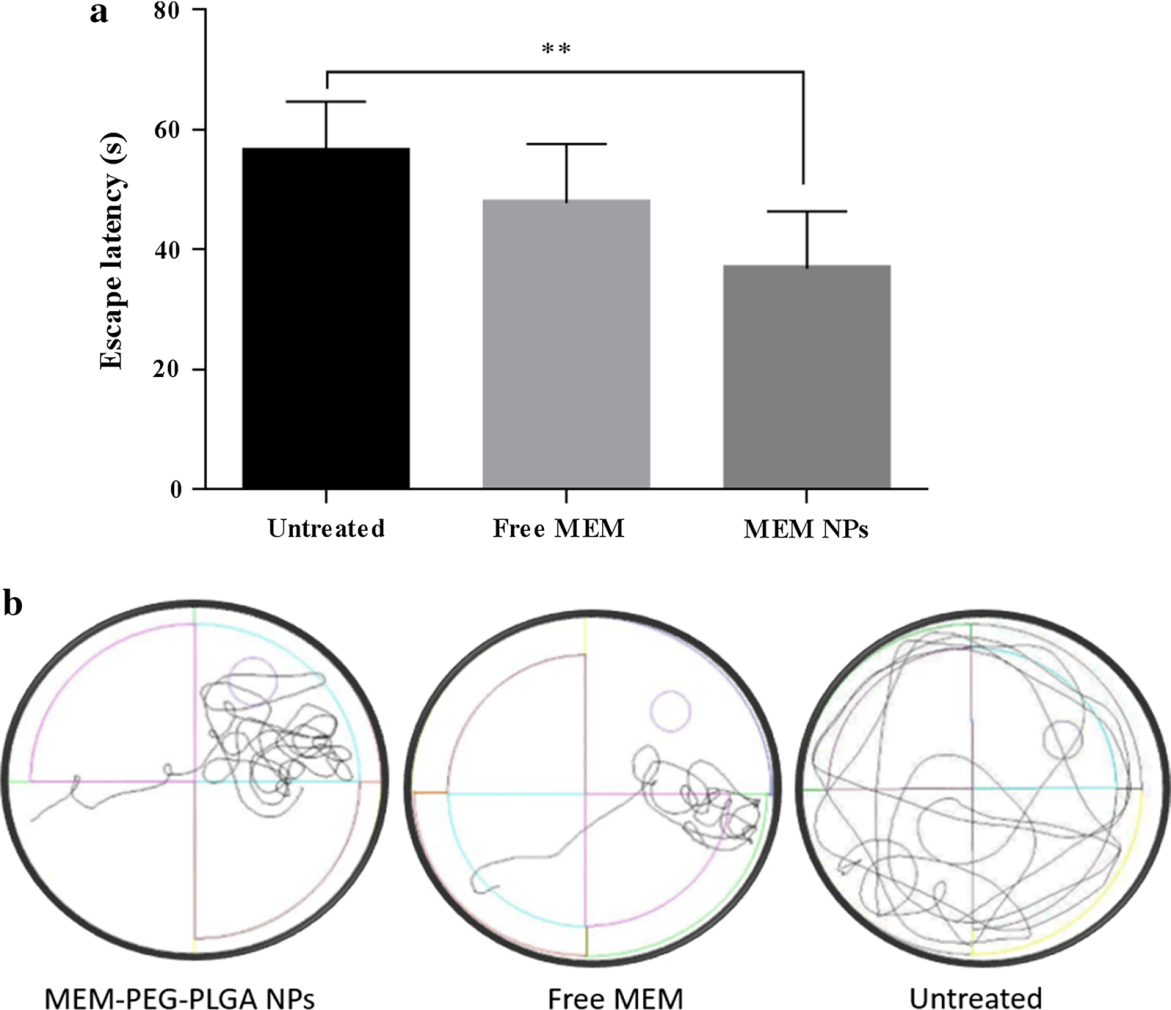
Morris water maze results on the probe trail of the APP/PS1 mice. **a** Escape latency and **b** representative swimming path of transgenic mice. Data represent mean ± SD; *p < 0.05, **p < 0.01, ***p < 0.001, ****p < 0.0001

The effects of MEM treatment on the animal’s behaviour were assessed with the MWM test (Fig. 8). The overall ANOVA for the training days revealed both a genotype (APP/PS1 against WT showed significant differences, p < 0.01) and a drug effect (treated vs untreated APP/PS1 mice) on mice spatial learning capacities. Escape latency on the test day results are shown in Fig. 8a. Untreated APP mice showed a significant increase on scape latency compared to MEM loaded NPs group (p  < 0.01). In addition, mice treated with NPs revealed an improvement on spatial learning memory compared with free MEM (no statistically significant differences). This indicates that the developed nanosystems constitute a suitable strategy for the delivery of drugs for AD.

As shown in Fig. 8b, MEM-loaded NPs groups followed a more direct path until platform, than the rest of transgenic groups. As expected, significant differences were obtained with APP/PS1 untreated group and MEM-loaded NPs (p < 0.01). Regarding time percentage in the platform quadrant (data not shown), APP/PS1 mice treated with MEM-loaded NPs presented an average of 37.22% of the time, whereas transgenic mice treated with MEM spend a 24.72% of the time revealing that oral MEM-loaded NPs restore cognition more effectively than the free drug.

### Immunohistochemistry

**Fig. 9:**
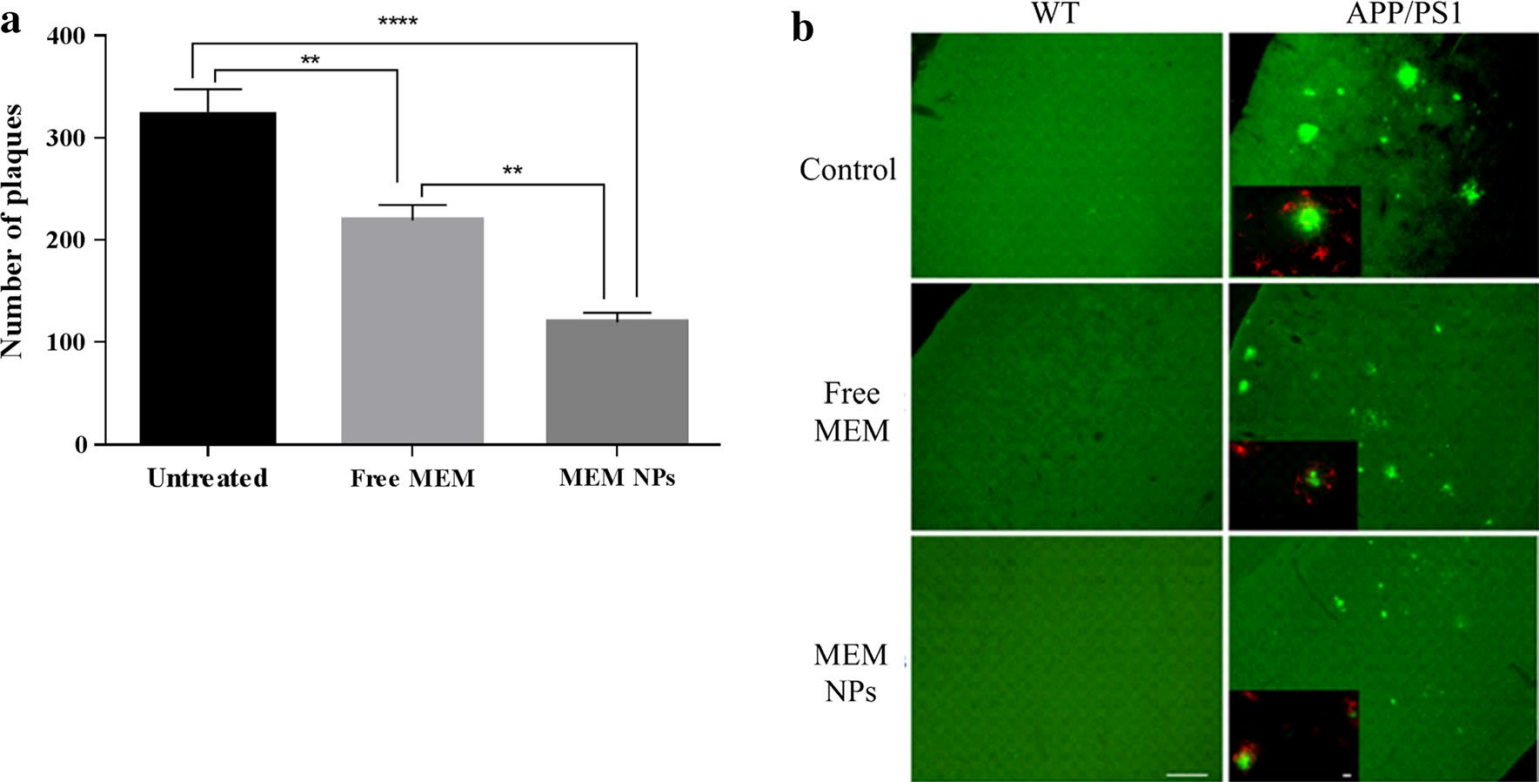
**a** Immunohistochemically (cortex) staining of amyloid plaques (green) and GFAP (red) of WT and APP/PS1 mice (untreated, MEM free and MEM loaded NPs). Bar reference equivalent to 100 µm. and **b** Amyloid plaques counting of APP/PS1 mice. Data represent mean ± SD; *p < 0.05, **p < 0.01, ***p < 0.001, ****p < 0.0001

The formation of Aβ plaques, which is a pathologic hallmark of AD, could be observed by Thioflavin˗S staining. Several studies confirmed that MEM decrease the number of amyloid plaques, therefore, histological studies to observe plaque development would be of great relevance. Figure 9a shows the results corresponding to amyloid plaques counting of APP/PS1 mice. WT groups did not develop β-amyloid plaques. APP/PS1 mice treated with NPs developed some plaques, but the number was significantly lower than those obtained for the rest of transgenic groups (p < 0.001 against untreated mice and p < 0.01 against MEM mice).

Figure 9b depicts the microscopic images after immunohistochemically staining of insoluble β-plaque development. APP untreated mice showed a greater plaque development. Moreover, plaques were surrounded by a high inflammatory state characteristic of AD. MEM–PEG–PLGA NPs groups showed fewer plaques and also inflammation degree was lower than the rest of transgenic groups [48]. These results are in agreement with behavioural assays, indicating that MEM restored cognition by decreasing insoluble amyloid plaques and the inflammatory response associated with AD.

## Conclusions

In this study, factorial design allowed to obtain NPs with an average size lower than 200 nm and PI < 0.1, characteristic of monodispersed systems, suitable to be absorbed by the gastrointestinal tract and release the drug across the BBB. The optimized formulation was obtained by adding 7.5 mg/ml of surfactant, a low polymer concentration and a high drug amount. NPs were washed by ultracentrifugation process and effective surfactant elimination was demonstrated both by XRD and FTIR since no PVA bands were observed in the NPs profile. This suggests that the surfactant only acts as an adjuvant in the NPs production, stabilizing the colloidal suspension and it is not entrapped in the polymer since it was effectively removed by centrifugation. This is an increase outcome since a high surfactant amount may induce toxicity by establishing an interconnected network with the polymer. MEM–PEG–PLGA NPs raised the T_g_ of the polymer, thus confirming the drug loading within the particles. Moreover, no evidence of strong bond or chemical interaction between drug and polymer was found. MEM–PEG–PLGA NPs did not show the drug melting and decomposition process observed in the physical mixture, confirming that the drug loaded into NPs was in the form of either a molecular dispersion or in a solid solution.

MEM–PEG–PLGA NPs showed to be physically stable upon 6 months storage both at 25 and 4 °C, being preferable 4 °C storage due to a slight NPs sedimentation process observed in the backscattering profile. The developed formulation presented a slow in vitro release profile at 37 °C against free drug both fitting to hyperbola equation. This could be due to a first fast drug release (burst effect) provided by the drug accumulated onto the NPs surface, followed by a released caused by the drug entrapped into the polymeric matrix.

The in vitro and in vivo results for brain drug levels showed clear evidence that the developed systems provide a sustained delivery of the drug into the target tissue. The developed colloidal systems increase drug amount into the target organ and confirm the suitability of the NPs for oral administration attributed to the bioadhesive polymer properties. Moreover, reduced administration frequency (on alternate days) demonstrated to be adequate to achieve brain therapeutic concentrations of drug. Behavioural and histological studies of APP/PS1 and WT mice treated with NPs in alternate days showed a better effect of NPs groups against free MEM treatment improving both learning capacities and β-amyloid brain plaques on APP/PS1 animals. This can be attributed to the sustained release obtained with MEM–PEG–PLGA NPs that provide a stable drug amount into the target organ.

In summary, MEM–PEG–PLGA NPs could be a promising alternative towards a better treatment of AD patients since NPs have demonstrated to be capable to provide a more effective treatment than free MEM.

## Additional file

**Additional file 1: Figure S1.** MEM-PLGA-PEG NPs transmission electron microscopy and size distribution obtained by dynamic light scattering. **Figure S2.** MEM thermogravimetric and differential thermal analysis. **Figure S3. A)** Experimental groups involved on the study, **B)** *In vivo* Timeline. **Figure S4.** Escape latency results of the Morris water maze test on the probe trail of the WT mice.

## Abbreviations

AD: Alzheimer’s disease
Aβ: β-amyloid
MEM: memantine
PLGA: poly(lactic-*co*-glycolic acid)
FDA: Food and Drug Administration
NPs: nanoparticles
PEG: poly(ethylene glycol)
PVA: polyvinyl alcohol
Z-AVE: mean average size
PI: polydispersity index
ZP: zeta potential
EE: encapsulation efficiency
TEM: transmission electron microscopy
P_e_: permeability across cell monolayer
APP/PS1: APPswe/PS1dE9 mice
