# Supplementary material for: Memantine loaded PLGA PEGylated nanoparticles for Alzheimer’s disease: in vitro and in vivo characterization
Source: J Nanobiotechnology. 2018 Mar 27;16:32. doi: 10.1186/s12951-018-0356-z (PMC5870370; doi:10.1186/s12951-018-0356-z)
Supplement: Supplementary file 1 — Additional file 1: Figure S1. MEM-PLGA-PEG NPs transmission electron microscopy and size distribution obtained by dynamic light scattering. Figure S2. MEM thermogravimetric and differential thermal analysis. Figure S3. A) Experimental groups involved on the study, B) In vivo Timeline. Figure S4. Escape latency results of the Morris water maze test on the probe trail of the WT mice. [file 12951_2018_356_MOESM1_ESM.pdf]

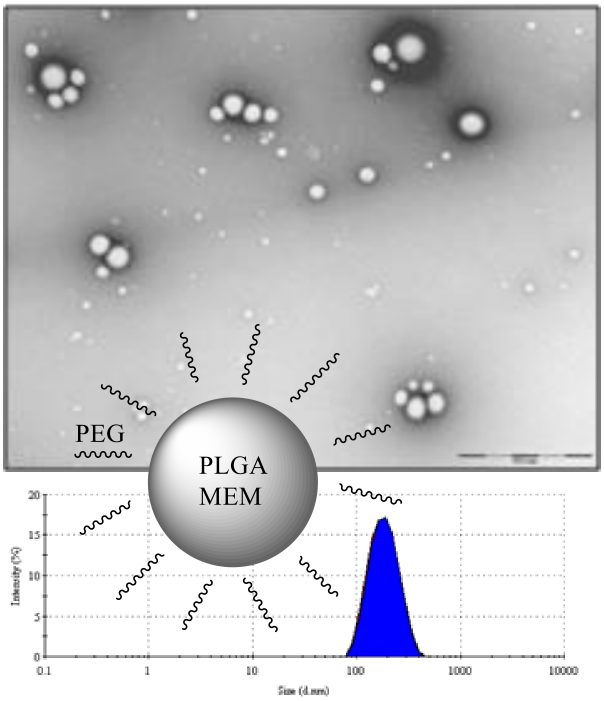

Figure S1. MEM-PLGA-PEG NPs transmission electron microscopy and size distribution obtained by dynamic light scattering.

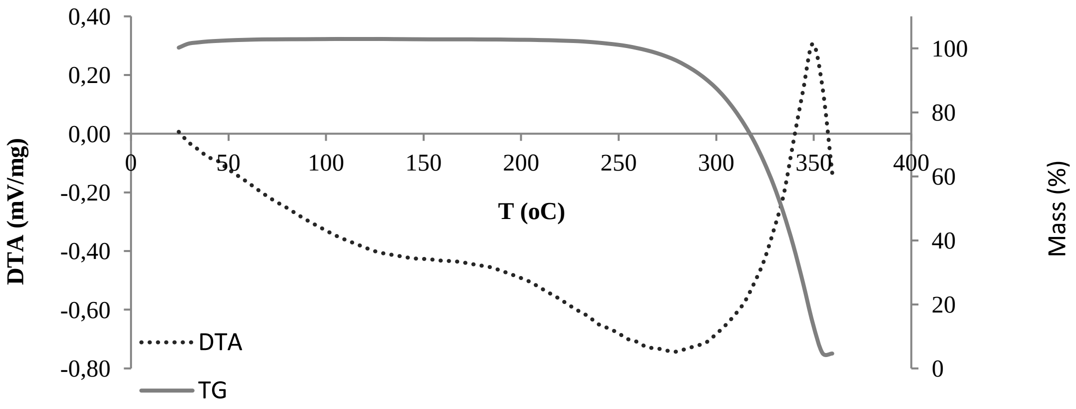

Figure S2. MEM thermogravimetric and differential thermal analysis.

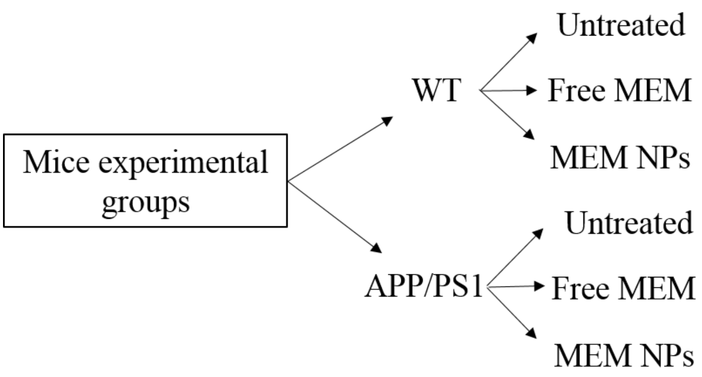

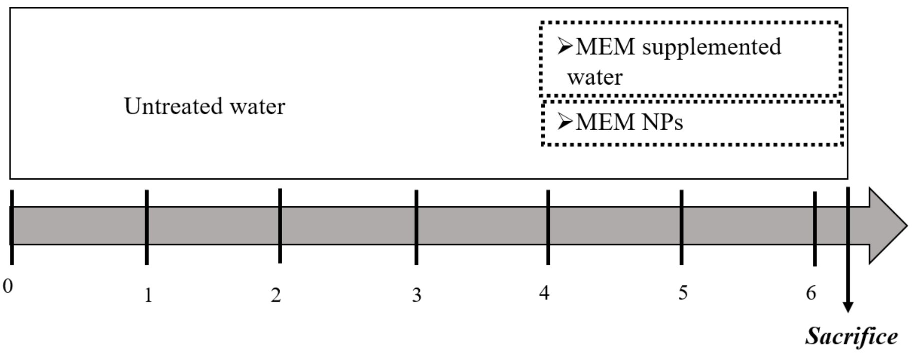

Figure S3. A) Experimental groups involved on the study, **B)** *In vivo* Timeline

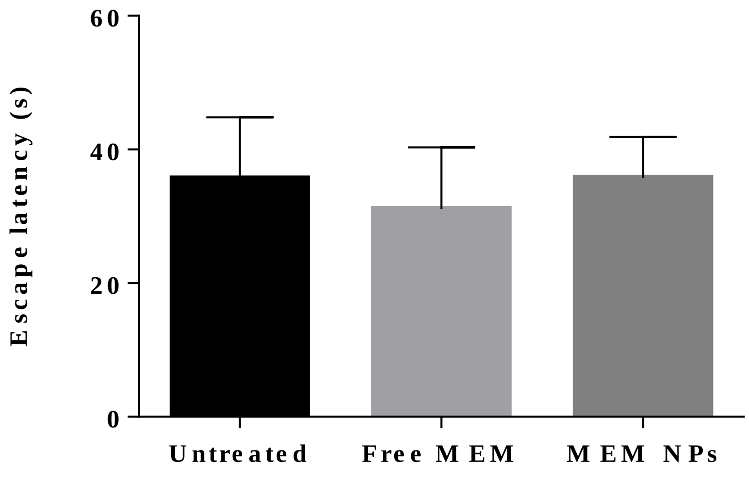

Figure S4. Escape latency results of the Morris water maze test on the probe trail of the WT mice.
